# Integration of HIV Status in Cancer Surveillance in South Africa: A Call for Action

**DOI:** 10.1002/cam4.71661

**Published:** 2026-02-26

**Authors:** Carole Metekoua, Tracey Wiggill, Tinashe Tombe‐Nyahuma, Yann Ruffieux, Judith Mwansa‐Kambafwile, Stanford Kwenda, Tafadzwa G. Dhokotera, Julia Bohlius, Eliane Rohner, Mazvita Muchengeti

**Affiliations:** ^1^ National Cancer Registry, National Health Laboratory Service Johannesburg South Africa; ^2^ Graduate School for Health Sciences University of Bern Bern Switzerland; ^3^ Institute of Social and Preventive Medicine University of Bern Bern Switzerland; ^4^ Division of Medical Microbiology and Immunology, Faculty of Health Sciences University of Stellenbosch Stellenbosch South Africa; ^5^ National Health Laboratory Service Cape Town South Africa; ^6^ Division of Epidemiology and Biostatistics, School of Public Health University of Witwatersrand Johannesburg South Africa; ^7^ School of Public Health University of Cape Town Cape Town South Africa; ^8^ Surveillance Data Warehouse National Institute of Communicable Diseases, National Health Laboratory Service Johannesburg South Africa; ^9^ Swiss Tropical and Public Health Institute Allschwil Switzerland; ^10^ University of Basel Basel Switzerland; ^11^ South African DSI‐NRF Centre of Excellence in Epidemiological Modelling and Analysis (SACEMA) Stellenbosch University Stellenbosch South Africa

**Keywords:** cancer, HIV, natural language processing, probabilistic record linkage, South Africa

## Abstract

**Background:**

Human immunodeficiency virus (HIV) increases the risk of developing cancer. We aimed to assign HIV status to cancers diagnosed in public laboratories recorded in the National Cancer Registry (NCR) in South Africa, guided by HIV counselling and testing guidelines.

**Methods:**

We used natural language processing to extract HIV‐related information from free‐text reports and probabilistic record linkage to match cancers diagnosed between 2004 and 2021 to HIV‐related tests from the National Health Laboratory Service Corporate Data Warehouse. We assigned HIV status based on the results of the HIV‐related tests and their timing relative to cancer diagnosis. We used descriptive statistics and logistic regression to examine HIV status documentation patterns and HIV prevalence in cancer patients.

**Results:**

Of the 496,517 cancers reported to the NCR, 41% (*n* = 203,937) had a documented HIV status. Documentation increased from 29% in 2004–2009 to 52% in 2016–2021. The odds of having a documented HIV status were 20% higher in females than in males and 16%–28% lower in other population groups compared with Black Africans. Patients with infection‐related cancers had almost threefold higher odds of having a documented HIV status than patients with infection‐unrelated cancers. Among cancer patients with documented HIV status, HIV prevalence was 75% for infection‐related and 32% for infection‐unrelated cancers.

**Conclusion:**

HIV status documentation among people with cancer has improved over time, but it is still suboptimal. Clinicians and pathologists in HIV endemic areas need to improve HIV ascertainment at cancer diagnosis and reporting to cancer registries to inform patient care and guide cancer control efforts.

## 1 Introduction

Human immunodeficiency virus (HIV)‐1 increases the risk of developing cancer in humans [1]. The carcinogenicity of HIV is explained by various mechanisms, including HIV‐induced immunosuppression, increased susceptibility to oncogenic viruses, genetic alterations, and chronic inflammation [2, 3, 4]. These processes also accelerate cell ageing, resulting in cancer occurring at an earlier age among people with HIV (PWH) compared to people without HIV [3]. Early in the HIV epidemic, the incidence rates of Kaposi sarcoma, non‐Hodgkin lymphoma, and cervical cancer were noted to be particularly high among people with uncontrolled HIV and, thus, were considered as AIDS‐defining cancers [5, 6, 7, 8]. With the availability of antiretroviral treatment (ART), the incidence rates of Kaposi sarcoma and non‐Hodgkin lymphoma [5, 6, 9] declined among PWH [5, 6, 9], but incidence rates of other cancers such as anal, lung, and colorectal cancer increased in this population [10]. Furthermore, PWH tend to have worse cancer survival than people without HIV [11, 12]. This could be a result of late cancer diagnosis [13], rapid cancer progression associated with immunosuppression [14], increased cytotoxicity with chemotherapy [11, 15], and an increased risk of developing second primary cancers [16, 17].

Changes in the HIV epidemic and ART access influence cancer epidemiology. Integrating HIV information in cancer surveillance systems—particularly in sub‐Saharan Africa, where HIV prevalence is high—is essential to monitor trends in cancer incidence and to guide resource allocation for cancer prevention and care. Although most cancer registries in HIV‐endemic areas are designed to document HIV status at cancer diagnosis, this information is often not systematically captured [18]. As a result, health care systems are limited in their ability to monitor and respond to changes in cancer incidence as the HIV epidemic evolves. In this study, we sought to assign HIV status to cancer diagnoses recorded in the National Cancer Registry (NCR) in South Africa using probabilistic record linkage (PRL) and natural language processing (NLP) methods. This work builds on previous efforts to obtain information on HIV status at cancer diagnosis in the NCR [19, 20], now incorporating additional data sources and drawing on the South African HIV counselling and testing guidelines [21].

## 2 Methods

### 2.1 Data Sources

We integrated data from the NCR and the National Health Laboratory Service (NHLS) to identify any information on HIV status for cancers diagnosed within public health facilities in South Africa.

#### 2.1.1 National Cancer Registry

The NCR is the legally mandated institution responsible for collecting and reporting on cancer in South Africa. Established as a voluntary pathology‐based cancer reporting system in 1986, the NCR became a legal entity in 2011 under Regulation 380 of the National Health Act (Act 61 of 2003) of South Africa. This regulation made reporting cancer diagnoses mandatory and required all cancers to be reported to the NCR. The NCR has three main registries, namely the pathology‐based, population‐based, and childhood cancer registry. This study only used data from the national pathology‐based registry.

The nation‐wide pathology‐based registry has been described in detail elsewhere [22]. Briefly, it is a passive cancer surveillance system that receives demographic information and free‐text laboratory reports of cancers diagnosed by histology, cytology, bone marrow aspirate, or trephine in public and private laboratories nationwide. These reports are assigned topography and morphology codes by coders guided by the International Classification of Diseases for Oncology, 3rd edition (ICD‐O‐3), to classify these reports [23]. The HIV status at cancer diagnosis is not currently reported to this registry.

#### 2.1.2 National Health Laboratory Service

The NHLS Corporate Data Warehouse (CDW) has been the primary source for building nationwide HIV cohorts in South Africa [24, 25]. The NHLS provides diagnostic pathology services in public health facilities, serving over 80% of South Africa's population. This is achieved through a network of more than 260 laboratories, each equipped with a laboratory information system to capture patient and test information. Information from all laboratories is consolidated into the CDW. For this study, we extracted data on HIV testing and monitoring from 2004 to 2021 from the NHLS‐CDW. All records with inconclusive HIV results were excluded. We also extracted all other laboratory test reports related to the cancer reports as identified through the NHLS‐CDW record linkage algorithm. This algorithm is a two‐stage record linkage process, starting with deterministic matching followed by probabilistic matching using national identification (ID) numbers, first name, surnames, and date of birth as linkage variables. We provide details of the implementation of this algorithm in Appendix S1.

### 2.2 Methods of Extracting Information on HIV Status

We used rule‐based NLP and PRL approaches on the extracted laboratory records to identify cancers with available information on HIV status.

#### 2.2.1 Natural Language Processing

We used a rule‐based NLP approach to extract the HIV status from cancer pathology reports at the NCR and other laboratory records of cancer patients within the NHLS‐CDW. This process was implemented in two steps. First, we used a keyword search to identify reports with HIV‐related terms (e.g., HIV, RVD, RDV, retrovirus, retroviral, anti‐retroviral, ART, HAART, CD4, immunosuppression, immuno‐compromised, immune‐deficiency, AIDS, etc.). Subsequently, we created a set of rules to classify reports with HIV‐related terms as HIV‐positive and HIV‐negative (Appendix S1). Reports without an HIV‐related term or a clear indication of HIV status were classified as HIV‐unknown.

#### 2.2.2 Record Linkage

We used the Python package Splink (version 4.0.6) [26] to probabilistically link records from the pathology‐based registry to the HIV‐related records from the NHLS‐CDW. Splink supports both supervised and unsupervised learning based record linkage methods. In this study, we applied supervised learning methods, using records with valid national ID numbers (13 digits) to train our linkage algorithm to calculate weighted similarity scores considering typographical errors within the first name, surname, sex, and date of birth. Records with a match probability score of 90% or more and records with the same national ID number were clustered as belonging to the same individual.

### 2.3 Assigning HIV Status at Cancer Diagnosis

**FIGURE 1:**
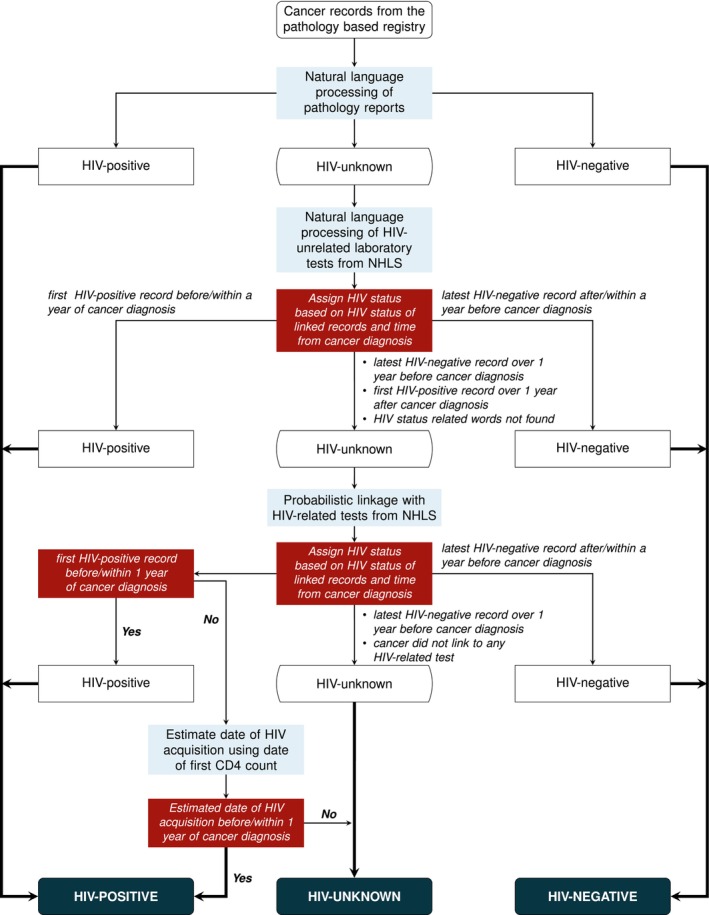
Process of identifying documented HIV status for cancer patients. HIV: human immunodeficiency virus; NHLS: national health laboratory service.

After extracting and linking HIV‐related information to cancer records, we assigned HIV status to each cancer record using a stepwise process, which is summarized in Figure 1. We used the HIV status information obtained through the NLP of pathology reports as a basis. Subsequently, for cancer records with unknown HIV status, we additionally used information from other NHLS‐CDW laboratory reports belonging to the same patient. If the HIV status remained unknown, we used information from the PRL of HIV‐related laboratory records. If cancer records linked to multiple HIV‐related laboratory reports, we selected one record per cancer for further processing using the following rules:

1. If all linked HIV‐related records indicated a negative HIV status, we selected the last result and the corresponding test date.
2. If all linked HIV‐related records indicated a positive HIV status, we selected the first result and the corresponding test date.
3. If there were conflicting HIV results on different days, we used the test result closest to the cancer diagnosis.
4. If HIV‐related records showed conflicting HIV information on the same day, we did not consider these records further.

We assigned a negative HIV status to cancers with a negative HIV test after cancer diagnosis or up to 1 year before cancer diagnosis. If the negative HIV test result was obtained more than 1 year before the cancer diagnosis, we considered the HIV status as unknown, aligning with South Africa's HIV counselling and testing policy, which recommends annual HIV testing among low‐risk individuals [21]. If a positive HIV test or an HIV monitoring test was recorded before or up to 1 year after cancer diagnosis, we considered the patient to be HIV‐positive at cancer diagnosis. If the first positive HIV test or HIV monitoring test was documented more than a year after the cancer diagnosis and a valid CD4 count was available, we estimated the approximate years an individual lived with HIV, considering the person's sex, using the formula below.

$$t=\frac{N-n}{80}$$

where *t* represents years with HIV infection; *n* is the first CD4 cell count; 80 is the approximate annual CD4 decay rate [27] and *N* is the reference average CD4 cell count among healthy individuals. The reference CD4 cell count used in this study was obtained from a study in rural KwaZulu‐Natal, South Africa, and was 833 cells/μL for females, 683 cells/μL for males, and 775 cells/μL for both combined [28]. Where sex was unknown in the cancer data, we used the average combined CD4 cell count as a reference.

We determined the estimated date of HIV acquisition by subtracting the estimated years with HIV infection (*t*) from the first CD4 cell count date. Where this date was before or within 1 year of cancer diagnosis, we assigned the cancer record a positive HIV status. If the date of the first HIV‐positive record and the estimated HIV acquisition date were more than 1 year after cancer diagnosis, we considered the HIV status to be unknown.

### 2.4 Statistical Analysis

We used frequencies and proportions to describe the characteristics of individuals diagnosed with cancer between 2004 and 2021. We used univariable and multivariable logistic regression models to identify demographic factors associated with HIV status documentation. We also described the distribution and completeness of HIV status at cancer diagnosis by calendar year, province, sex, age group, population/racial/ethnic group, and cancer types using frequencies and proportions. We described HIV documentation for nine infection‐related and nine infection‐unrelated cancers individually.

## 3 Results

### 3.1 Study Population

The public health laboratories reported 496,517 pathologically confirmed cancers to the South African NCR between 2004 and 2021. Table 1 provides a summary of the characteristics of these cancer patients. Cancer diagnoses were evenly distributed across calendar periods. Gauteng province accounted for the highest proportion of diagnoses (32%, *n* = 159,009), followed by Western Cape (22%, *n* = 108,274) and Eastern Cape (12%, *n* = 60,838). Most cancer patients were female (59%, *n* = 292,164) and Black African (68%, *n* = 329,933), with a median age at cancer diagnosis of 56 years (interquartile range [IQR]: 43, 67). Two‐thirds of cancers were infection‐unrelated (69%, *n* = 344,780).

**TABLE 1:** Characteristics of individuals diagnosed with cancer.

Characteristic	Number (%)
Total	496,517 (100%)
Age group (years)	
< 15	10,026 (2.1%)
15–24	9700 (2.0%)
25–34	38,319 (7.9%)
35–44	71,595 (14.8%)
45–54	95,731 (19.7%)
55–64	115,042 (23.7%)
≥ 65	144,743 (29.8%)
Missing	11,361
Sex	
Female	292,164 (58.9%)
Male	203,675 (41.1%)
Missing	678
Population group	
Black African	329,933 (68.7%)
White	73,521 (15.3%)
Coloured	65,287 (13.6%)
Indian/Asian	11,833 (2.5%)
Missing	15,943
Calendar period	
2004–2009	163,780 (33.0%)
2010–2015	168,343 (33.9%)
2016–2021	164,394 (33.1%)
Cancer type	
Infectionrelated	151,737 (30.6%)
Infection‐unrelated	344,780 (69.4%)
Province of cancer diagnosis	
Eastern Cape	60,838 (12.4%)
Free State	31,841 (6.5%)
Gauteng	159,009 (32.4%)
KwaZulu‐Natal	55,765 (11.4%)
Limpopo	21,478 (4.4%)
Mpumalanga	23,460 (4.8%)
North West	17,882 (3.6%)
Northern Cape	11,638 (2.4%)
Western Cape	108,274 (22.1%)
Missing	6332

### 3.2 Sources of HIV Status

Using each data source independently, we assigned HIV status at cancer diagnosis to 8% (*n* = 41,943) of cancer patients from pathology reports, 13% (*n* = 64,316) from NHLS‐CDW laboratory reports, and 37% (*n* = 185,563) from PRL and imputation of HIV acquisition date. Combining methods and data sources, we were able to assign a known HIV status to 41% (*n* = 203,937) of cancer patients. For 21% of cancer patients with an assigned HIV status (*n* = 41,943), HIV information was extracted from pathology reports, for 12% (*n* = 23,652) from other NHLS‐CDW laboratory reports, and for 68% (*n* = 138,342) from PRL and imputation of HIV acquisition date.

### 3.3 Patterns of HIV Documentation

**FIGURE 2:**
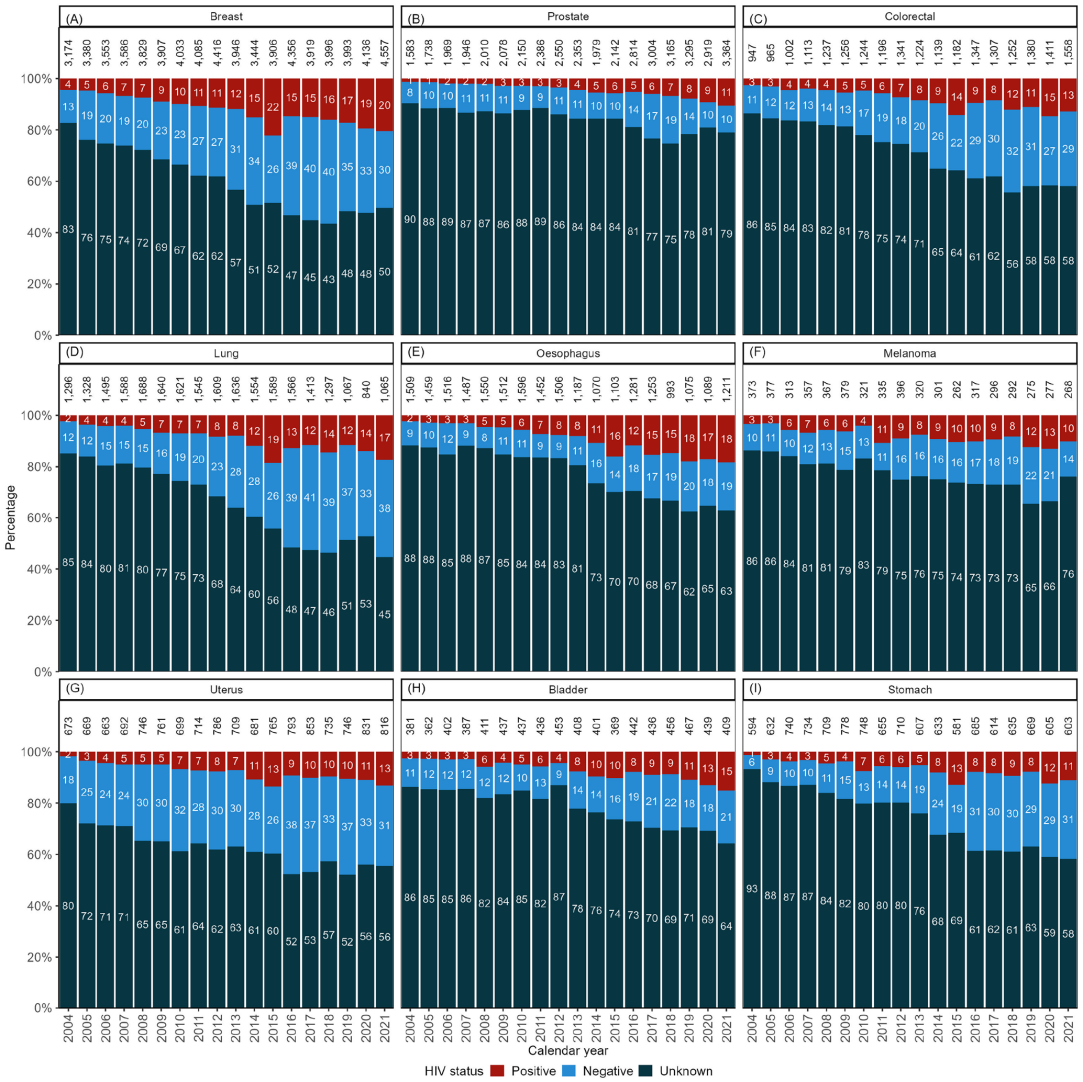
Distribution of HIV status by calendar year for the most common infection‐unrelated cancers: (A) Breast, (B) Prostate, (C) Colorectal, (D) Lung, (E) Oesophagus, (F) Melanoma, (G) Uterus, (H) Bladder, and (I) Stomach. The values displayed in the bars are percentages (%), and the total per bar may slightly exceed 100% due to rounding. Numbers above bars denote case counts per year.

**FIGURE 3:**
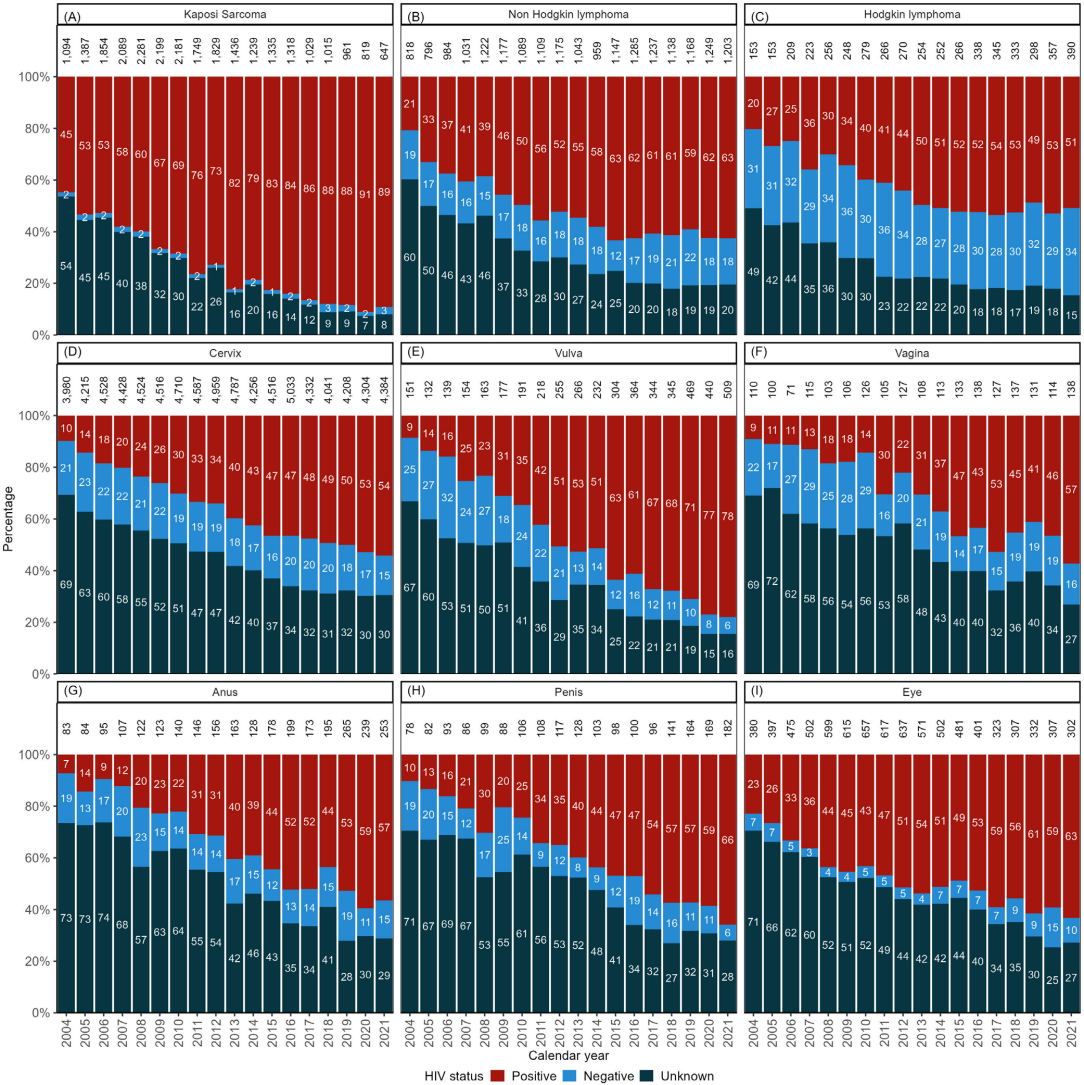
Distribution of HIV status by calendar year for the most common infection‐related cancers: (A) Kaposi sarcoma, (B) Non‐Hodkgin lymphoma, (C) Hodgkin lymphoma, (D) Cervix, (E) Vulva, (F) Vagina, (G) Anus, (H) Penis, and (I) Eye. The values displayed in the bars are percentages (%), and the total per bar may slightly exceed 100% due to rounding. Numbers above bars denote case counts per year.

The documentation of HIV status increased from 29% in 2004–2009 to 52% in 2016–2021 (Table 2), with HIV documentation being substantially less complete for infection‐unrelated cancers (Figure 2) than for infection‐related cancers (Figure 3). Higher levels of HIV documentation were observed among female, young or middle‐aged, and Coloured or Black African individuals (Table 2). The odds of having an HIV status documented increased substantially over time and were more than threefold higher in 2016–2021 than 2004–2009 (adjusted odds ratio [aOR] 3.20, 95% confidence interval [CI] 3.14–3.25). HIV documentation was more common among female than male cancer patients, although this association was attenuated in the adjusted analysis (aOR 1.16, 95% CI 1.15–1.18). Cancer patients aged 65 years or older were least likely to have information on HIV status captured (Table 3). In the adjusted analysis, all other population groups had 16%–28% lower odds of documented HIV status than Black Africans. Patients with infection‐related cancers had almost threefold higher odds of having a documented HIV status (aOR 2.81, 95% CI 2.77–2.86) than those with infection‐unrelated cancers. We also found provincial variation, with patients in the Western Cape, Free State, Northern Cape, and North West provinces being more likely to have information on HIV status captured.

**TABLE 2:** Documentation of HIV status.

	Known HIV status			Unknown
	Negative	Positive	Total	
	(*N* = 99,464)	(*N* = 104,473)	(*N* = 203,937)	(*N* = 292,580)
Age group (years)				
< 15	4412 (80.5%)	1069 (19.5%)	5481 (54.7%)	4545 (45.3%)
15–24	2481 (46.3%)	2877 (53.7%)	5358 (55.2%)	4342 (44.8%)
25–34	5081 (20.4%)	19,867 (79.6%)	24,948 (65.1%)	13,371 (34.9%)
35–44	11,591 (25.8%)	33,394 (74.2%)	44,985 (62.8%)	26,610 (37.2%)
45–54	22,325 (46.6%)	25,546 (53.4%)	47,871 (50.0%)	47,860 (50.0%)
55–64	27,331 (65.3%)	14,515 (34.7%)	41,846 (36.4%)	73,196 (63.6%)
≥ 65	26,168 (79.6%)	6700 (20.4%)	32,868 (22.7%)	111,875 (77.3%)
Missing	75	505	580	10,781
Sex				
Female	62,044 (46.9%)	70,201 (53.1%)	132,245 (45.3%)	159,919 (54.7%)
Male	37,389 (52.2%)	34,195 (47.8%)	71,584 (35.1%)	132,091 (64.9%)
Missing	31	77	108	570
Population group				
Black African	50,741 (36.1%)	89,765 (63.9%)	140,506 (42.6%)	189,427 (57.4%)
White	20,631 (79.8%)	5229 (20.2%)	25,860 (35.2%)	47,661 (64.8%)
Coloured	23,228 (78.4%)	6387 (21.6%)	29,615 (45.4%)	35,672 (54.6%)
Indian/Asian	2286 (74.6%)	780 (25.4%)	3066 (25.9%)	8767 (74.1%)
Missing	2578	2312	4890	11,053
Calendar period				
2004–2009	26,260 (54.9%)	21,599 (45.1%)	47,859 (29.2%)	115,921 (70.8%)
2010–2015	32,497 (46.0%)	38,097 (54.0%)	70,594 (41.9%)	97,749 (58.1%)
2016–2021	40,707 (47.6%)	44,777 (52.4%)	85,484 (52.0%)	78,910 (48.0%)
Cancer type				
Infection‐related	23,393 (25.3%)	69,039 (74.7%)	92,432 (60.9%)	59,305 (39.1%)
Infection‐unrelated	76,071 (68.2%)	35,434 (31.8%)	111,505 (32.3%)	233,275 (67.7%)
Province of cancer diagnosis				
Eastern Cape	9343 (49.8%)	9426 (50.2%)	18,769 (30.9%)	42,069 (69.1%)
Free State	8063 (48.3%)	8616 (51.7%)	16,679 (52.4%)	15,162 (47.6%)
Gauteng	24,636 (39.0%)	38,506 (61.0%)	63,142 (39.7%)	95,867 (60.3%)
KwaZulu‐Natal	4763 (29.6%)	11,344 (70.4%)	16,107 (28.9%)	39,658 (71.1%)
Limpopo	1648 (23.1%)	5481 (76.9%)	7129 (33.2%)	14,349 (66.8%)
Mpumalanga	1988 (18.0%)	9041 (82.0%)	11,029 (47.0%)	12,431 (53.0%)
North West	3054 (36.2%)	5376 (63.8%)	8430 (47.1%)	9452 (52.9%)
Northern Cape	3044 (60.8%)	1959 (39.2%)	5003 (43.0%)	6635 (57.0%)
Western Cape	42,362 (75.1%)	14,030 (24.9%)	56,392 (52.1%)	51,882 (47.9%)
Missing	563	694	1257	5075

**TABLE 3:** Factors associated with a documented HIV status.

	Unadjusted OR (95% CI)	Adjusted OR (95% CI)
Age group (years)		
< 15	4.10 (3.94–4.28)	4.70 (4.49–4.92)
15–24	4.20 (4.03–4.38)	4.33 (4.13–4.54)
25–34	6.35 (6.20–6.51)	5.31 (5.16–5.46)
35–44	5.75 (5.64–5.87)	4.84 (4.74–4.95)
45–54	3.40 (3.34–3.47)	3.21 (3.15–3.28)
55–64	1.95 (1.91–1.98)	1.92 (1.89–1.96)
≥ 65	Ref.	Ref.
Sex		
Male	Ref.	Ref.
Female	1.53 (1.51–1.54)	1.16 (1.15–1.18)
Population group		
Black African	Ref.	Ref.
White	0.73 (0.72–0.74)	0.72 (0.71–0.74)
Coloured	1.12 (1.10–1.14)	0.84 (0.82–0.86)
Indian/Asian	0.47 (0.45–0.49)	0.84 (0.80–0.88)
Calendar period		
2004–2009	Ref.	Ref.
2010–2015	1.75 (1.72–1.77)	1.91 (1.88–1.94)
2016–2021	2.62 (2.59–2.66)	3.20 (3.14–3.25)
Cancer type		
Infection‐unrelated	Ref.	Ref.
Infection‐related	3.26 (3.22–3.30)	2.81 (2.77–2.86)
Province of cancer diagnosis		
Gauteng	Ref.	Ref.
Eastern Cape	0.68 (0.66–0.69)	0.86 (0.84–0.88)
Free State	1.67 (1.63–1.71)	2.26 (2.19–2.32)
KwaZulu‐Natal	0.62 (0.60–0.63)	0.55 (0.54–0.57)
Limpopo	0.75 (0.73–0.78)	0.74 (0.71–0.76)
Mpumalanga	1.35 (1.31–1.38)	0.89 (0.86–0.92)
North West	1.35 (1.31–1.40)	1.59 (1.53–1.65)
Northern Cape	1.14 (1.10–1.19)	1.91 (1.83–2.00)
Western Cape	1.65 (1.62–1.68)	2.70 (2.65–2.76)

Abbreviations: CI, confidence interval; OR, odds ratio.

### 3.4 HIV Prevalence by Patient Characteristics and Cancer Type

Among cancer patients with a documented HIV status, HIV prevalence increased from 45% in 2004–2009 to 54% in 2010–2015, before declining slightly to 52% in the most recent period. Prevalence was higher among female (53%) than male (48%) cancer patients and peaked in the 25–34 age group (80%). Black African cancer patients had the highest HIV prevalence (64%) among all population groups. HIV prevalence in patients with infection‐related cancers (75%) was more than twice that of patients with infection‐unrelated cancers (32%). Generally, the HIV prevalence among patients with infection‐unrelated cancers increased over time. However, most of these patients with infection‐unrelated cancers remained HIV‐negative over time, except for oesophageal and prostate cancer, where HIV prevalence among those with a documented HIV status reached approximately 50% (Figure 2). HIV prevalence among patients with infection‐related cancers also increased over time, with anogenital human papillomavirus (HPV)‐related cancers shifting from occurring predominantly among HIV‐negative to HIV‐positive patients (Figure 3).

## 4 Discussion

We assigned HIV status to cancer records from public health facilities within the pathology‐based NCR in South Africa using NLP, PRL, and HIV counselling and testing guidelines and identified factors associated with the documentation of HIV status. We were able to assign a known HIV status to 41% of 496,517 cancers captured between 2004 and 2021. HIV status documentation improved over time, particularly for infection‐related cancers. However, the HIV status was unknown for two‐thirds of patients with infection‐unrelated cancers. Female, Black African or Coloured, and young or middle‐aged cancer patients were particularly likely to have their HIV status recorded and, among those with a documented HIV status, to have a positive HIV status.

To our knowledge, this is the largest study assessing and improving HIV documentation within a nationwide cancer registry. We integrated various data sources and used PRL and NLP methods to obtain information on HIV status. Also, we considered HIV counselling and testing guidelines to refine our algorithms. However, several limitations need to be considered. HIV testing primarily occurs in primary health clinics not linked to the NHLS‐CDW, which might lead to an underrepresentation of HIV‐negative results in the databases used for this study. HIV‐positive results are more likely to be included in our study, given that HIV monitoring tests are done at NHLS laboratories that feed into the NHLS‐CDW. The actual documentation of HIV status may be higher than reported, as our PRL approach may have missed some true matches, and we lacked access to clinical notes that could have provided additional confirmation. Moreover, the results might not be equally representative for all population groups, as more than two‐thirds of White individuals are covered by medical aid [29] and may access health care services in private instead of public health facilities. Our results are likely to be most representative for the general Black African population in South Africa, among whom over 90% access health care services in the public health sector [29].

Data on HIV documentation and prevalence in cancer registries are scarce. Previous studies conducted at hospital‐based cancer registries in different sub‐Saharan African countries reported a large variation in HIV documentation proportions, ranging from 22% at the Kamuzu Central Hospital in Malawi to 92% at the Charlotte Maxeke Johannesburg Academic Hospital in South Africa, with 71% at the Uganda Cancer Institute and 86% at the Parirenyatwa Group of Hospitals in Zimbabwe [18, 30]. HIV documentation in our study was substantially lower than the 92% reported for the hospital‐based registry in South Africa. This difference likely reflects incomplete ascertainment of HIV status when relying on PRL and NLP, whereas hospital‐based registries capture HIV status more directly through clinical record review. Furthermore, the completeness of HIV documentation at an academic hospital in Johannesburg may not necessarily be representative of HIV documentation practices across all hospitals in the country. In sub‐Saharan Africa, HIV prevalence among cancer patients with documented HIV status is generally reported to be three to four times higher than in the general population [18, 31]. Accordingly, in our study, compared with the 2022 South African general population, HIV prevalence was approximately three times higher in female cancer patients (53% vs. 20%) and four times higher in male cancer patients (48% vs. 11%) [32].

HIV counselling and testing were launched in South Africa in 2000 as a voluntary programme within public health facilities. In 2004, the programme was expanded to include stand‐alone voluntary counselling and testing at non‐medical sites in community settings. Client‐initiated and provider‐initiated HIV counselling and testing were introduced in all public health facilities in 2010, prioritizing groups such as women attending antenatal care and family planning, children attending integrated management of childhood illnesses, and individuals diagnosed with tuberculosis and sexually transmitted infections [21]. In 2016, HIV counselling and testing were restructured and renamed as HIV testing services to put greater emphasis on providing pre‐ and post‐test counselling, ensuring people are linked to appropriate HIV care services, and improving coordination with laboratories to ensure accurate test results [21]. This shift led to an improved uptake of HIV testing services in the country [21]. National HIV surveys in South Africa showed that the proportion of self‐reported known HIV status in the general population increased from 49% in 2008 to 67% in 2017 [33], which may have, in turn, contributed to the increase in documentation of HIV status from 29% in 2004–2009 to 52% in 2016–2021 in our study. However, despite the general increase in self‐reported HIV testing, HIV documentation among cancer patients remains suboptimal. The lower proportion of known HIV status among cancer patients compared to the general population may be partly explained by cancer patients being, on average, older. Over half of all cancer diagnoses in our study occurred in adults aged 55 years or older, and this age group is less likely to be tested for HIV than younger individuals [34]. In contrast, most of the general population in South Africa is aged 15–49 years [35], and this age group is more likely to test for HIV regularly due to higher perceived risk [34]. Other reasons for lower HIV documentation in our study include potential overreporting of HIV testing in surveys [36, 37] and limited integration of clinical information in laboratory reports. Furthermore, HIV test results from point‐of‐care tests at primary health clinics do not require a laboratory facility and might be missed, especially if the results are negative. This is because negative results are not followed by HIV monitoring tests, which are typically performed in a laboratory and captured in the NHLS‐CDW. Moreover, health care providers may be less likely to mention a negative HIV status in a report, as it is often considered less clinically relevant than a positive HIV status, which may directly inform patient care.

We found that female, Black African, and younger cancer patients were more likely to have an HIV status documented. This is in line with another study showing that people aged ≥ 60 years in Malawi and Zimbabwe were less likely to have a documented HIV status than those < 40 years [18]. These patterns reflect the demographic distribution of the HIV epidemic. Notably, in South Africa's general population, HIV prevalence is particularly high among young and middle‐aged, female Black Africans [33]. This indicates that the awareness of population‐level HIV prevalence influences the health care providers' decisions to test for HIV and document a cancer patient's HIV status. Furthermore, women tend to interact with the health care system more often than men through reproductive, maternal, and child health services, increasing the likelihood of having an HIV test performed and documented, while gender norms and stigma discourage health care seeking among men [38]. Additionally, certain cancer types such as Kaposi sarcoma, lymphomas, and other infection‐related cancers increase health care providers' index of suspicion for HIV infection, resulting in improved documentation of HIV status for patients with these cancer types. This suggests that HIV testing in cancer care is still driven by clinical suspicion and not necessarily aligned with provider‐initiated counselling and testing guidelines [21].

The improvement in ART coverage over time has significantly extended the life expectancy of PWH [39]. As a result, PWH are increasingly developing cancers common in the general population [40]. Additionally, HIV induces accelerated cellular ageing [41], which may increase the likelihood of developing age‐related cancers at a younger age. This could partly explain the increase in HIV prevalence observed across both infection‐unrelated and infection‐related cancers over time. For HPV‐related anogenital cancers like cervical, vulvar, vaginal, penile, and anal cancers, we documented a shift from patients being predominantly HIV‐negative to HIV‐positive. This aligns with results from the US‐based HIV/AIDS Cancer Match study showing that for vulvar cancer the standardized incidence ratios comparing women with HIV to the general population increased over time [42]. HIV infection doubles the risk of acquiring HPV and reduces the rate of HPV clearance by about half [43]. The elevated risk of HPV persistence combined with the longer life expectancy among PWH may contribute to the substantial increase in HIV prevalence among patients with HPV‐related cancers in South Africa.

The evolving HIV epidemic is impacting cancer trends, necessitating evidence‐based resource allocation for integrated care. Limited HIV status documentation in cancer reports, as noted in this study, negatively impacts effective planning for health care systems in HIV endemic areas to address the double burden of cancer and HIV. Therefore, clinicians and pathologists in HIV endemic areas should ensure that all cancer patients are tested for HIV and that results are reported to cancer registries alongside cancer incidence to inform resource allocation and patient care.

## 5 Conclusion

In conclusion, we used NLP and PRL on three distinct data sources to ascertain HIV status at cancer diagnosis for cancers reported to the NCR by public health laboratories in South Africa. While documentation of HIV status improved for most cancers over the years, the proportion of cancer patients with documented HIV status remained low, particularly for infection‐unrelated cancers. Individuals with a higher perceived risk of acquiring HIV were more likely to have their HIV status documented than individuals at lower risk. Integration of HIV status in cancer care and surveillance is required to enable effective evidence‐based resource allocation and improve patient care in high HIV burden settings.

## Author Contributions

**Carole Metekoua:** conceptualization, data curation, formal analysis, methodology, visualization, writing – original draft, writing – review and editing. **Tracey Wiggill:** methodology, supervision, writing – review and editing. **Tinashe Tombe‐Nyahuma:** data curation, writing – review and editing. **Yann Ruffieux:** methodology, writing – review and editing. **Judith Mwansa‐Kambafwile:** writing – review and editing. **Stanford Kwenda:** writing – review and editing. **Tafadzwa G. Dhokotera:** writing – review and editing. **Julia Bohlius:** funding acquisition, writing – review and editing. **Eliane Rohner:** conceptualization, funding acquisition, methodology, supervision, writing – original draft, writing – review and editing. **Mazvita Muchengeti:** conceptualization, funding acquisition, methodology, writing – review and editing.

## Funding

Research reported in this publication was supported by the US National Institutes of Health (NIH)'s National Institute of Allergy and Infectious Diseases; the Eunice Kennedy Shriver National Institute of Child Health and Human Development; the National Cancer Institute; the National Institute of Mental Health; the National Institute on Drug Abuse; the National Heart, Lung, and Blood Institute; the National Institute on Alcohol Abuse and Alcoholism; the National Institute of Diabetes and Digestive and Kidney Diseases; and the Fogarty International Center under Award Number U01AI069924, by the Swiss National Science Foundation (Grant Numbers 10004150 and 320030_169967), and the Swiss Cancer Research foundation (KFS‐5415‐08‐2021). The content is solely the responsibility of the authors and does not necessarily represent the official views of the funders.

## Ethics Statement

This study was conducted under the South African HIV Cancer Match study ethics approval (M240458) and the ethical waiver (W‐CBP‐230118‐01) granted by the University of the Witwatersrand's Human Research Ethics Committee. The waiver allows the National Cancer Registry to fulfil its legislative mandate for cancer surveillance in South Africa and to support related research. The waiver permits researchers at the registry to publish epidemiological analyses based on routinely collected data without requiring individual informed consent or additional ethical approvals. All procedures in this study adhered to the ethical principles outlined in the Declaration of Helsinki.

## Conflicts of Interest

The authors declare no conflicts of interest.

## Supporting information


**Appendix S1:** Information on record linkage approach and regular expressions used to extract information on HIV status.

## Data Availability

The data used for natural language processing and probabilistic record linkage contains personal identifiers and cannot be shared with the public. Nonetheless, the National Cancer Registry data with the assigned HIV status supporting the findings of this study are available upon reasonable request through the National Health Laboratory Service Academic Affairs and Research Management System (https://aarms.nhls.ac.za).
