# Supplementary material for: Integration of HIV Status in Cancer Surveillance in South Africa: A Call for Action
Source: Cancer Med. 2026 Feb 26;15(3):e71661. doi: 10.1002/cam4.71661 (PMC12945709; doi:10.1002/cam4.71661)
Supplement: Supplementary file 1 — Appendix S1: Information on record linkage approach and regular expressions used to extract information on HIV status. [file CAM4-15-e71661-s001.docx]

**Title: Integration of HIV Status in Cancer Surveillance: A Call for Action**

**SUPPLEMENTARY MATERIAL**

Supplementary Text 1: Record linkage within the National Health Laboratory Service Corporate Data Warehouse (NHLS-CDW)

Supplementary Table 1: Regular expressions used to extract information on HIV status

**Supplementary Text 1**: Record linkage within the National Health Laboratory Service Corporate Data Warehouse (NHLS-CDW)

The NHLS-CDW incorporates a record linkage algorithm to assign a unique identifier to all laboratory results obtained for an individual over time. Briefly, the NHLS-CDW team first cleans the linkage variables and then uses a two-stage record linkage process, starting with deterministic matching followed by probabilistic matching. The cleaning process includes removing titles from names, replacing missing values by “unknown”, and validating the format of the national identification (ID) numbers. Subsequently, deterministic matching checks for exact matches on valid national ID, then on first name, surname, and date of birth combined. Records from academic hospitals are also deterministically matched using folder numbers and facility codes. Records that match deterministically are then deduplicated by retaining the most frequent first name, surname, and date of birth combination before applying probabilistic matching algorithms. Within the probabilistic algorithm, records with the same first name, first letter, and date of birth are grouped together for further comparisons in terms of first name, surname and date of birth. A weighted similarity probability score with the weights of 40% for surname, 40% for first name, and 20% for date of birth, is computed for records within the same groups. Records are considered as belonging to the same person if they have a weighted probability score of 0.90 and above and are assigned a unique patient identifier. Finally, the most representative demographic attributes (first name, surname, and date of birth) for each linked record group are used as patient details in specialised tables within the NHLS-CDW.

**Supplementary Table 1:** Regular expressions used to extract information on HIV status

| **HIV Status** | **Regular expression patterns** |
| --- | --- |
| Positive | \b(  (RVD\|HIV\|RDV\|retro(?:virus\|viral)?)[\s\W]*(patient\|client\|pos\|positive\|positief\|react(ive\|ing)\|affirmed\|defaulted\|on[\s\W]*(rx\|treatment\|therapy)\|patient\|reaktief\|\(\+\)[\s\W]*\|\+[\s\W]*\|\+ve[\s\W]*) \|  HAART\s* \|  ART[\s\W]*s?\s* \| ARV[\s\W]*s?\s* \|  RVD[\s\W]*R\s* \| RVD[\s\W]*\(R\)\s* \|  anti[\s\W]*retro(?:viral\|virus)?[\s\W]*(treatment\|therapy) \|  RETRO(?:virus\|viral)?[\s\W]*disease[\s\W]*(patient\|client\|pos\|positive\|positief\|react(ive\|ing)\|affirmed\|defaulted\|on[\s\W]*(RX\|TREATMENT\|THERAPY)\|patient\|reaktief\|\(\+\)[\s\W]*\|\|\+[\s\W]*\|\+ve[\s\W]*) \|  (with\|has\|known\|from)[\s\W]*(RVD\|RDV\|HIV\|AIDS\|HIV[\s\W]*\/[\s\W]*AIDS\|RETRO(?:virus\|viral)?[\s\W]*disease) \|  defaulted[\s\W]*(art\|arts\|arv\|anti[\s\W]*retro(?:viral\|virus)?) \|  (RVD\|HIV\|RDV\|retro(?:virus\|viral)?)[\s\W]*\+ \|  diagnos[a-zA-Z]*[\s\W]*(RVD\|HIV\|RDV\|retro(?:virus\|viral)?) \|  positi(ve\|ef)[\s\W]*(RVD\|HIV\|RDV\|retro(?:virus\|viral)?) \|  (RVD\|HIV\|RDV\|retro(?:virus\|viral)?)[\s\W]*NEWLY[\s\W]*diagnosed \|  IMMUN(O\|E)[\s\W]*COMPROMISED \|  reactive\s*for\s*RETROVIRAL\s*DISEASE \|  \n(rvd\|HIV\|retro(?:virus\|viral)?)\s*disease\.?\n \|  \n(rvd\|HIV\|retro(?:virus\|viral)?)[\s\W]*\n \|  HISTORY\s*OF\s*(RVD\|HIV\|retroviral\s*disease\|RDV) \|HIV\s*VIRAL\s*LOAD \|  background\s*(RVD\|HIV\|retroviral\s*disease\|RDV) \|  is\s*(RVD\|HIV\|retroviral\s*disease\|RDV)\s*positive \|cd4 \|HIV\s*viral\s*load\s* \|  (advanced\|uncontrolled\|untreated)\s*(HIV\|RVD\|RDV\|retro(?:virus\|viral)?)\|  seropositive \|stage\s*(I\|II\|III\|IV\|1\|2\|3\|4)\s*HIV \|HIV\s*stage\s*(I\|II\|III\|IV\|1\|2\|3\|4)\|HIV-1/2\s*AB/AG\s*ELISA\s*RESULT\s*POSITIVE \|  diagnosis\s*of\s*(RVD\|HIV\|retroviral\s*disease) \| sero[\s\W]pos[a-zA-Z]*[\s\W]* \|  (RVD\|HIV\|RDV\|retro(?:virus\|viral)?)[\s\W]*(associated\|related)  )\b |
| Negative | \b(  (RVD\|HIV\|RDV\|retro(?:virus\|viral)?)[\s\W]*(neg[a-zA-Z]*\|non[\s\W]*reactive\|un[\s\W]*reactive\|\(\-\)\|\-[\s\W]*\|\-ve[\s\W]*\|NR)\|  neg[a-zA-Z]*[\s\W]*(RVD\|HIV\|RDV\|retro(?:virus\|viral))\|  NEGATIVE[\s\W]*FOR[\s\W]*(RVD\|RDV\|HIV\|retro(?:virus\|viral)?)\|  (retro(?:virus\|viral)?\s+disease)[\s\W]*(neg[a-zA-Z]*\|non[\s\W]*reactive)[\s\W]*\|  no[\s\W]*(RVD\|RDV\|HIV\|retro(?:virus\|viral))[\s\W]*disease[\s\W]*\|  un[\s\W]*reactive[\s\W]*(RVD\|HIV\|RDV\|retro(?:virus\|viral))  (un\|non)[\s\W]*reactive[\s\W]*for[\s\W]*(RVD\|HIV\|RDV\|retro(?:virus\|viral))[\s\W]*\|  (un\|non)[\s\W]*reactive[\s\W]*for[\s\W]*(RVD\|HIV\|RDV\|retro(?:virus\|viral))\s+disease[\s\W]*\|  sero[\s\W]*neg[a-zA-Z]*[\s\W]*\|HIV-1/2\s*AB/AG\s*ELISA\s*RESULT\s*negative  )\b |
| Unknown | \b(  (RVD\|RDV\|HIV\|RETRO(virus\|viral))[\s\W]*(UNKNOWN\|status[\s\W]*UNKNOWN\|  disease[\s\W]*status[\s\W]*UNKNOWN\|disease[\s\W]*status[\s\W]*is[\s\W]*(not[\s\W]*KNOWN\|UNKNOWN)\|  status[\s\W]*is[\s\W]*(not[\s\W]*KNOWN\|UNKNOWN)\|unkown)\|  no[\s\W]*(RVD\|RDV\|HIV\|RETRO(virus\|viral))\|  declines[\s\W]*(RVD\|HIV)\|  (RVD\|HIV\|retroviral\s*disease)\s*(unknown\|STATUS\|UNKNOWN)  )\b |
